# Using an Ontology to Derive a Sharable and Interoperable Relational Data Model for Heterogeneous Healthcare Data and Various Applications

**DOI:** 10.1055/a-1877-9498

**Published:** 2022-12-03

**Authors:** Christina Khnaisser, Luc Lavoie, Benoit Fraikin, Adrien Barton, Samuel Dussault, Anita Burgun, Jean-François Ethier

**Affiliations:** 1 GRIIS, Université de Sherbrooke, Sherbrooke, Canada; 2 IRIT-MELODI, CNRS, Toulouse, France; 3 INSERM UMRS 1138 Team 22, Université de Paris, Paris, France

**Keywords:** heterogeneous data, information, data integration, ontologies, management

## Abstract

**Background** A large volume of heavily fragmented data is generated daily in different healthcare contexts and is stored using various structures with different semantics. This fragmentation and heterogeneity make secondary use of data a challenge. Data integration approaches that derive a common data model from sources or requirements have some advantages. However, these approaches are often built for a specific application where the research questions are known. Thus, the semantic and structural reconciliation is often not reusable nor reproducible. A recent integration approach using knowledge models has been developed with ontologies that provide a strong semantic foundation. Nonetheless, deriving a data model that captures the richness of the ontology to store data with their full semantic remains a challenging task.

**Objectives** This article addresses the following question: How to design a sharable and interoperable data model for storing heterogeneous healthcare data and their semantic to support various applications?

**Method** This article describes a method using an ontological knowledge model to automatically generate a data model for a domain of interest. The model can then be implemented in a relational database which efficiently enables the collection, storage, and retrieval of data while keeping semantic ontological annotations so that the same data can be extracted for various applications for further processing.

**Results** This article (1) presents a comparison of existing methods for generating a relational data model from an ontology using 23 criteria, (2) describes standard conversion rules, and (3) presents *O n t o R e l a* , a prototype developed to demonstrate the conversion rules.

**Conclusion** This work is a first step toward automating and refining the generation of sharable and interoperable relational data models using ontologies with a freely available tool. The remaining challenges to cover all the ontology richness in the relational model are pointed out.

## Introduction

A large volume of healthcare data is generated daily in many contexts. In some of them, data are heavily fragmented across multiple heterogeneous systems. To provide care, manage a hospital, run clinical trials, or help clinicians make better decisions, the core need is to understand an individual's data through a broad scope (e.g., billing relies on integrating diagnosis and treatment data to sum the costs). Consequently, the semantic and structural reconciliation for the secondary use of data is a challenging task that can lead to incorrect interpretation if not correctly automated and verified.

This challenge is compounded by the fact that health data are highly dependent on contextual information. For example, a diabetes code could represent a working diagnosis, an established diagnosis, a family history of diabetes, or even a reason to ask for a test. Thus, to operate safely, the processes rely not only on accessing data from multiple systems, but also on the semantic of the data. 1 The semantics include the nature of the element (e.g., being a patient is a role, the same individual can have the role of a patient when going to the emergency for a fracture fixed, but also the role of a physician when coming to work as an orthopaedic surgeon to fix a fracture) and explicit relationships with other elements (e.g., a diagnosis of diabetes is about an individual who is my mother).

Accordingly, this article addresses the question: How to design a sharable and interoperable data model for storing heterogeneous healthcare data and its semantic to support various applications?

In many heterogeneous environments, a sharable and interoperable data model based on a knowledge model has been demonstrated as a valid approach to decipher the structure and identify relevant data elements to be extracted or combined in a semantically interoperable sound way. 2 Furthermore, to control data integrity while manipulating a large amount of data, a relational database (RDB) is an appropriate system to store data due to its embedded access control, transaction management, data integrity control, efficiency, and performance. 3 However, conventional techniques (entity-relationship, star schema, object-oriented techniques) do not provide sufficient semantic or contextual information for efficient use of a data model in a heterogeneous environment and ensure reliable reuse of data outside a restricted field of its original application. 1 4 Moreover, data generated across different systems (e.g., health ministries, pharmacies, clinics, hospitals) are often stored in RDBs, but their semantic is rarely documented or updated. In other words, conventional techniques do not offer adequate construct to allow the unification of data with their semantic. Thus, the multiple stages of data processing and the exchanges that take place along the way can result in incomplete semantics of the extracted dataset. Therefore, a domain with such fragmentation and multiple data providers but with very low error tolerance needs a new approach to encapsulate the data and their semantics into the same structure that can be shared and reused for multiple applications. Healthcare is one such domain, as life and death decisions will be based on these data.

Various approaches exist 5 to access or integrate data from multiple sources. These approaches use different kinds of inputs. Source-driven approaches use the data source's structure. Requirement-driven approaches use the user requirements for a specific application. Hybrid approaches combine the data structure of the sources and user requirements. Finally, knowledge-driven approaches derive the data model from the knowledge model of the domain. The knowledge-driven approach is arguably the most appropriate for the healthcare domain. Indeed, using a source-driven approach is an arduous task because data sources are structured according to the underlying application, and the addition of new sources will often lead to changes to the model. Moreover, using requirements is impractical because of the diversity and evolution of user requirements. Finally, neither of these last two approaches gives access (by themselves) to explicit semantic as the predicates associated with a data model are always dependent on the source application or a set of user requirements at a specific point in time. On the contrary, a knowledge-driven approach can provide a more stable data model in which the semantic is made explicit 1 and can serve various applications.

Biomedical formal ontologies have been used to formalize biomedical knowledge (e.g., genetics with Gene Ontology and the ontology for biomedical investigations, a reference in the field). They have been successfully used to support many data-related processes such as data integration, data annotation, and classification in many projects. 6 7 8 Developing biomedical formal ontologies is becoming more and more mature, and an international community, the OBO Foundry, is engaged in creating, coordinating, and maintaining these biomedical ontologies. 9 A realist ontology can be defined as “ *classes that denote exclusively entities that exist objectively in reality and [whose] definitions adhere to strict criteria to ensure that the classes are reusable in other ontologies while preserving their ontological commitment.* ” 10 Thus, these ontologies can be used as a knowledge model to describe a specific domain of discourse objectively and formally. Therefore, deriving a relational data model directly from an ontology can be hypothesized to be the best way to leverage heterogeneous data and their semantic while ensuring integrity and concurrency access for various applications. Specifically, a relational data model generated from an ontology ensures explicit axiomatization of the model structure enabling access to data and their associated semantic. Moreover, ontologies enable interactions with the database by referring to knowledge rather than ad hoc table, field structure and naming. Consequently, data storage, formulation and calculation of queries can be more systematic and reliable.

The presented method differs from other approaches involving mapping ontologies and databases described in the literature. Namely, the goal is not to create an ontology from source databases or to store the ontology in a database. Instead, the ontology *objectively* captures the domain's semantic rather than the database's structure. In this way, the resulted RDB reflects the ontology and explicitly ties it with the data. Also, the method does not convert data into a triple store (Resource Description Framework [RDF] triples) as it would not work in the use case of interest where the actors require a relational endpoint for data integrity reasons. Nevertheless, if a process benefits from transforming data from an RDB into a triple store, the work presented here would still greatly facilitate and simplify this task.

This article presents a method for deriving a relational data model from an ontology using conversion rules that covers more ontological constructs compared with existing methods including axiom complexity reduction rules, an implementation, OntoRelα, a freely available prototype used to demonstrate the conversion rules on various ontologies, a use case, a brief survey of existing methods, and a highlight of the contributions and remaining challenges.

## Objective

Many limitations remain with the existing methods to derive a sharable and interoperable relational data model based on a knowledge model for a heterogeneous environment. More specifically, every relational construct must be derived from a specific ontological construct uniformly in the same way, to reach uniformity and consistency through data integrity checks. Moreover, the generated data model must not reflect decisions based on specific query requirements to allow data usage outside the source database's scope or a specific project. Consequently, the standardization and automation of the conversion through a set of rules increase the relevance and consistency of the derived relational data model and reduce the risk of errors and ambiguities that might be unnoticeably introduced by choices influenced by undocumented aspects of the designers' reality. The objective is to address the gaps found in the literature regarding ontological construct coverage, including complex axioms found in biomedical ontologies, and to offer a publicly available implementation. Thus, this article presents an advanced conversion method and accessible implementation that handles more ontological constructs and complex axioms.

## Method

An ontology is constructed using classes, individuals, axioms, properties (object properties and data properties), cardinality restrictions, datatypes, and annotations. 11 12 A relational model is constructed by a set of relations defined by attributes (pairs of a unique name and a datatype), tuples, constraints, and functions. 13 14 Both models share common foundations: the set theory and the first-order logic. Thus, at least in part, a conversion from one to the other is possible.

The distinctive characteristic of the presented method is that it is automated using uniform and consistent conversion rules to capture the richness of the ontology. As a result, the derived relational data model is shareable and interoperable and is practical for storing heterogeneous health data for various applications. The conversion rules must include an axiom complexity reduction process and generate a highly normalized relational schema to minimize data redundancy and potential contradictions. The following illustrates a set of conversion rules with examples presented by ontology constructs. A complete example can be found in Appendix C.

### Conversion Rules

**Fig. 1:**
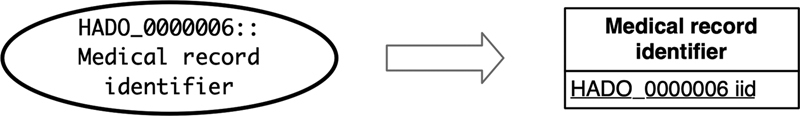
Class conversion example (the ellipse represents a class described using the short Internationalized Resource Identifiers [IRI] and a label; the rectangle represents a relation described using the relation name and the list of attributes).

*Class [C]* is a set of individuals (also known as. instances). A class is converted to a relation that includes an Individual Identifier attribute ( *classIri_iid:iid_type* ) used as a primary key (see Fig. 1 ). Each value of *iid* uniquely identifies an individual. The class *Thing* (the superclass of all classes) may be converted to a relation with one attribute, the *iid* . Thus, the relation *Thing* contains all individual identifiers of the database. This conversion makes it possible to define an independent artificial key to index individuals.

**Fig. 2:**
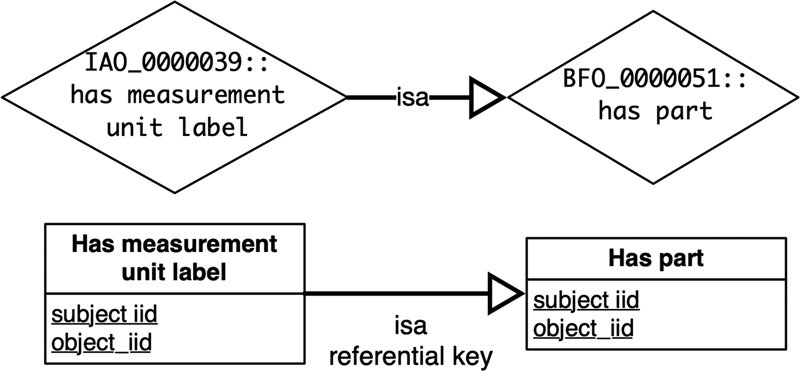
Object property conversion example.

*Object Property [op]* links two individuals. An object property is converted to a relation including two *iid* attributes (see Fig. 2 ): the subject ( *subject_iid:iid_type* ) and the object ( *object_iid:iid_type* ). This conversion allows direct access to the links between the data of two relations and allows to store links that are not explicitly specified by an axiom of the ontology.

**Fig. 3:**
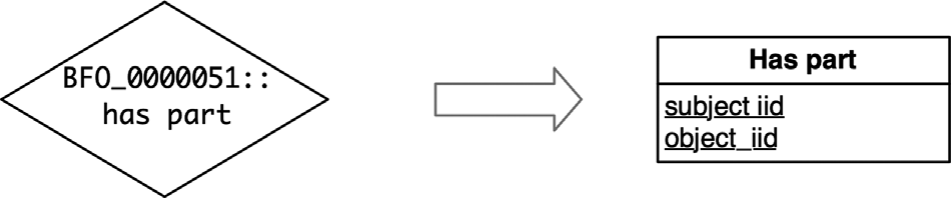
Property inheritance axiom conversion example.

**Fig. 4:**
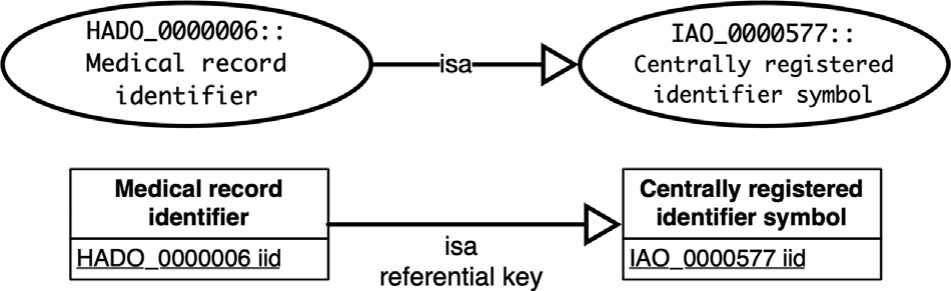
Class inheritance axiom conversion example.

*Property inheritance axiom [p _0_ ⊑ p _1_ ]* defines an inheritance between two properties. A property inheritance axiom is converted into an “isa” referential key from the sub-property relation to the super-property relation (see Fig. 3 ). A referential key in a relational data model links two relations (see Fig. 4 ). This conversion ensures data integrity by preserving the taxonomy of the properties.

*Class inheritance axiom [C _0_ ⊑ C _1_ ]* defines an inheritance between two classes. A class inheritance axiom is converted into an “isa” referential key from the subclass relation to the superclass relation. This conversion ensures data integrity by preserving the taxonomy of the classes.

**Fig. 5:**
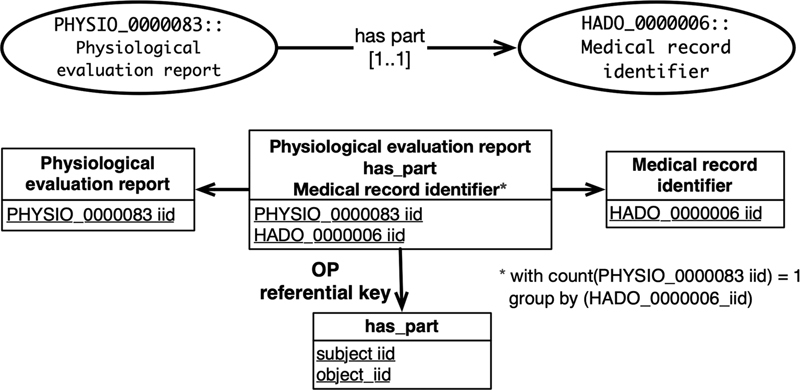
Class association axiom conversion example.

*Class association axiom [C _0_ op qt C _1_ ]* defines an association between individuals belonging to two classes according to an object property and a quantifier. An object property [op] links two individuals. A quantifier [qt] is an interval of integers that specifies the association's cardinality in which individuals can participate. A class association axiom is converted into an association relation (a relation related to two other relations) with two attributes, the primary key of both associated class relations (see Fig. 5 ). The primary key of the association relation is composed of both attributes. Moreover, three object property referential keys are defined from the association relation to the primary key of each associated class relation and property relation. Also, if qt.min > 0 and qt.max ∈ N, a quantification constraint is defined to check the number of individuals according to the quantification specified in the axiom. This conversion controls data integrity by ensuring that all the tuples in the relation have the same predicate with respect to the quantifier.

*Datatype [D]* is a constrained set of values. A datatype is converted into an SQL user-defined type reused to specify the data attributes uniformly. For example, the OWL xsd:String is converted to CREATE DOMAIN “xsd:String” AS TEXT (in the PostgreSQL syntax). This conversion maintains the datatype definition uniformly across all the relations.

**Fig. 6:**
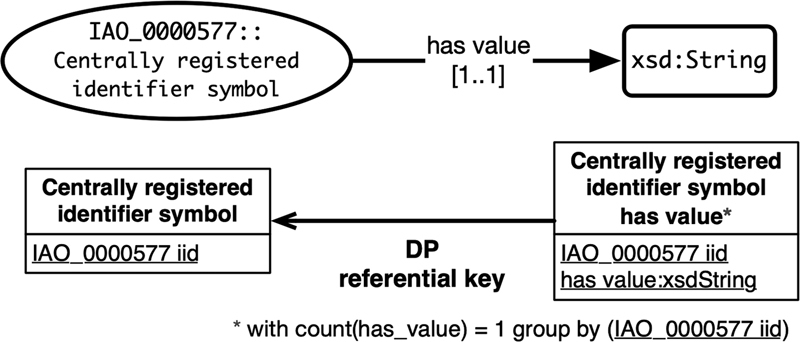
Data association axiom conversion example.

*Data association axiom [C _0_ dp qt D _1_ ]* defines an association between each individual of a class [C _0_ ] and a value of a datatype [D _1_ ] according to a data property [dp] and a quantifier. A data property links an individual to a value. A data association axiom is converted into a data relation with two attributes (see Fig. 6 ): the primary key of the class relation and a data attribute. The data relation's primary key is composed of both attributes. One data property referential key is defined from the data relation to the class relation. This conversion controls data integrity by avoiding redundancy and missing information.

**Fig. 7:**
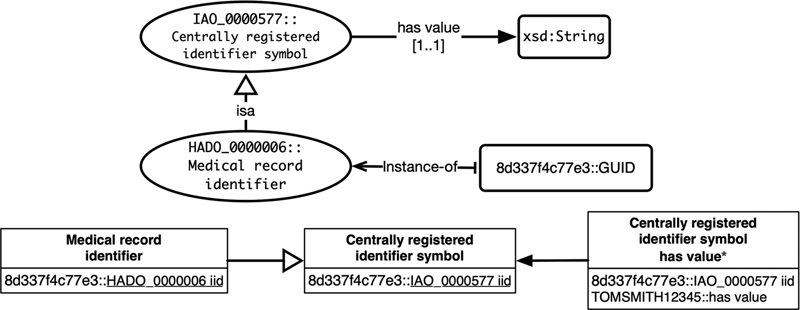
Individual conversion example.

*Individual [I]* is an entity of the modeled reality. An individual is converted into a tuple inserted into the proper relations according to the class of the individual (see Fig. 7 ). It is strongly recommended that iid attribute values be automatically generated (e.g., Globally Unique Identifier [GUID]), allowing independent individual indexing and automatically propagating values into the relations.

*Annotation* describes an aspect of an ontological construct with a text in a specific natural language. An annotation is used to document the database and to provide multiple access interfaces in different languages using views. A definition annotation is converted to SQL comment so they can be integrated into the RDBMS catalog (if the target RDBMS supports it). This conversion allows the documentation of relational constructs within the schema.

### Axiom Complexity Reduction

An ontological axiom can be defined using different expressions that are logically equivalent. It follows that a considerable number of cases must be considered when dealing with axioms such as complex axioms. A complex axiom is an expression formed with multiple expressions connected using a conjunction (AND) or a disjunction (OR) operation. Complex axioms must be reduced into a simpler form to ensure a rigorous conversion into predictable and consistent relational constructs. Thus, axiom complexity reduction functions are defined. The complexity reduction consists of generating a set of simple axioms from a set of complex axioms. To simplify an axiom, each expression in the complex axiom is replaced by a new uniquely named class derived according to a set of rules. To simplify an ontology, the process is applied recursively until all axioms are simplified. Each expression in an axiom is syntactically analyzed using the abstract grammar and reduced recursively according to reduction rules until a set of simple axioms is reached. More formal details can be found in Appendix B.

Here is a concrete didactic example of a subset of the PDRO, 15 an ontology about drug prescriptions, with one complex axiom (in bold).

**Fig. 8:**
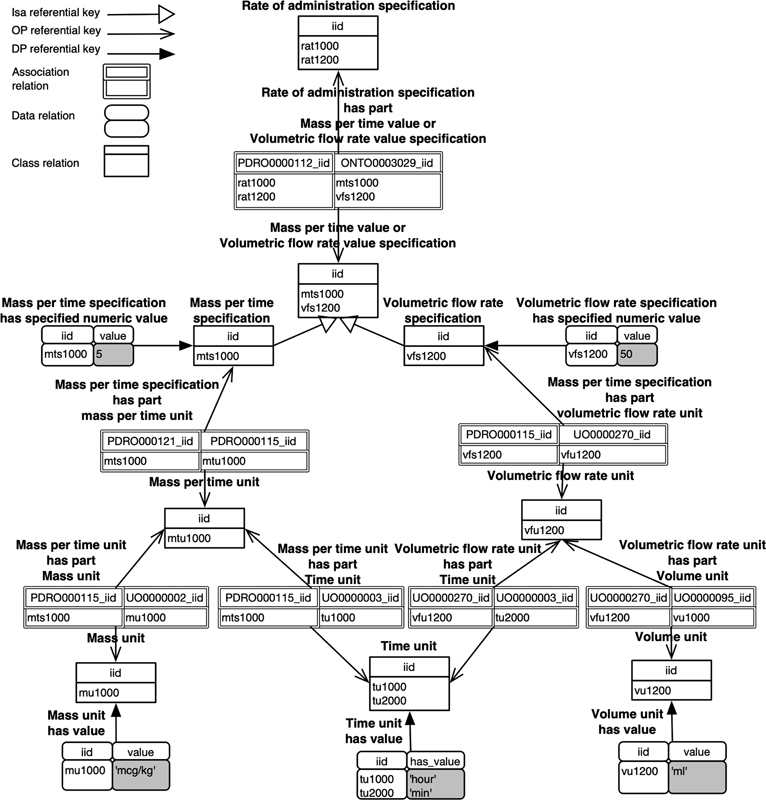
Example of relational schema with data.

After the axiom complexity reduction, the resulted ontology is as follows:

which can be derived into the following relational schema (see Fig. 8 ).

## Results

To illustrate the feasibility and the applicability of the presented method, a prototype, OntoRelα, was developed. As an input, OntoRelα takes an OWL ontology and some configuration files. It outputs an RDB script for the PostgreSQL database, a list of warnings to notify the user of problems in the conversion process, a mapping catalog between each ontological construct and relational construct (named OntoRelCat), and a normalized version of the ontology after the axiom complexity reduction. The resulted RDB scripts can be executed on PostgreSQL v9.6 + . A small example of the human body mass from the physiological measurement ontology is presented in Appendix C.

**Fig. 9:**
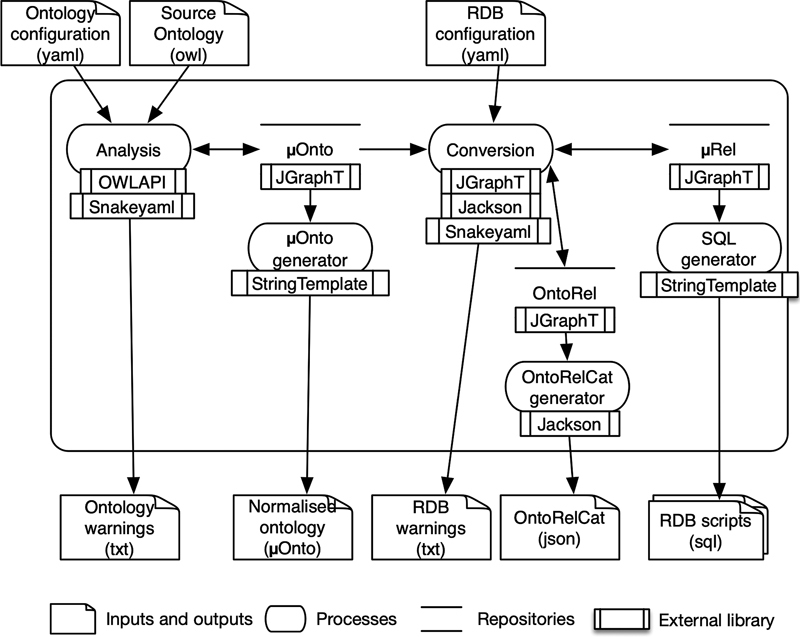
Data flow diagram of OntoRelα.

The method is implemented through multiple processes (see Fig. 9 ):

- The analysis process creates an instance of the normalized ontology model (µOnto) using a source ontology and an ontology configuration file that parameterizes the process.
- The µOnto generator process generates a normalized ontology with reduced axioms formalized according to the µOnto language.
- The conversion process converts an instance of µOnto into an ontological–relational model (OntoRel) according to the relational model (µRel) using the RDB configuration file that parameterizes the process.
- The OntoRelCat generator process generates the definitions of construct in the OntoRel to build a human and machine-readable mapping catalog.
- The SQL generator process generates a set of SQL scripts to build the RDB.

Many open-source libraries were used to implement the processes and the internal data structure: OWLAPI 5.1 ^a^ to load and analyze the ontology in OWL format; JGraphT ^b^ to create graphs for the ontology and the database; Snakeyaml ^c^ to analyze the configuration files in YAML format; StringTemplate ^d^ for code generation and Jackson ^e^ to generate JSON files for OntoRelCat.

The prototype was tested with various ontologies (especially the Genotype Ontology, Fanconi Anemia Ontology, Ontology of Adverse Events, and the Prescription of Drugs Ontology) of different sizes. Also, the resulted relational data model was used in different use cases of the Quebec SPOR Support Unit and the SPOR Canadian data platform. 16 The prototype and detailed results, including the RDB scripts, can be found on GitHub: https://github.com/OpenLHS/OntoRela .

## Use Case

Software used to record clinical data generally does not provide explicit semantic to enable secondary use of data without ambiguity. In addition, there is currently no national standard for data exchange across institutions and provinces in Canada. This use case illustrates the context of drug prescriptions where researchers or physicians need to extract information about drug and laboratory prescriptions to make appropriate follow-up or identify inappropriate or missing prescriptions. Users are faced with many challenges, including and not limited to heterogeneity in levels of generality in drug administration and dispensing specification, homonymy, and dosing instructions. 15 Moreover, merging data of drugs and laboratory results stored in different databases is not a straightforward task.

PDRO, 15 an ontology about drug prescriptions, is used to illustrate this use case with examples. OntoRelα generates the relational data model, and the database can be populated from data sources using Extract–Load–Transform processes or mediation systems. In this way, one query suffices to obtain needed data at various levels of generality without the need to explore each source separately to understand the content.

Consider parts of two drug prescriptions termed “Drug administration specification” (DAS) and “Drug dispensing specification” (DDS) written on a paper or stored in a nonstandard database:

- (DAS1) “Metoprolol 50 mg PO bid” instructs taking Metoprolol 50 mg per mouth (PO) twice a day (bid),
- (DDS1) “Apo-Metoprolol 50 mg tab, 1 tab PO bid” instructs taking one tab of Apo-Metoprolol 50 mg per mouth twice a day.

With DAS1, most clinicians would intend to prescribe the active ingredient “Metoprolol” rather than a specific drug product name manufactured by a specific company like “Apo-Metoprolol” because all pharmacies do not have in inventory every possible brand. However, DDS1 does refer to such a specific pharmaceutical product. Moreover, even strings that are identical in their composition and order of characters may have different meanings. For example, “Metoprolol” in DAS1 would usually refer to any drug product containing metoprolol or to the active ingredient metoprolol itself, although it might refer to the generic drug product branded with the name “Metoprolol” in some cases. Moreover, the information on the prescribed drug may differ from the dispensed drug, and more often, databases do not distinguish between them (between the DAS and the DDS).

**Fig. 10:**
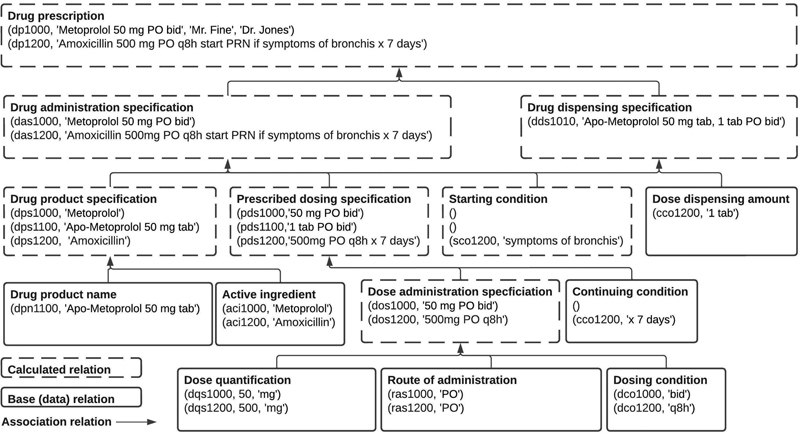
OntoRel with data as a graph.

**Fig. 11:**
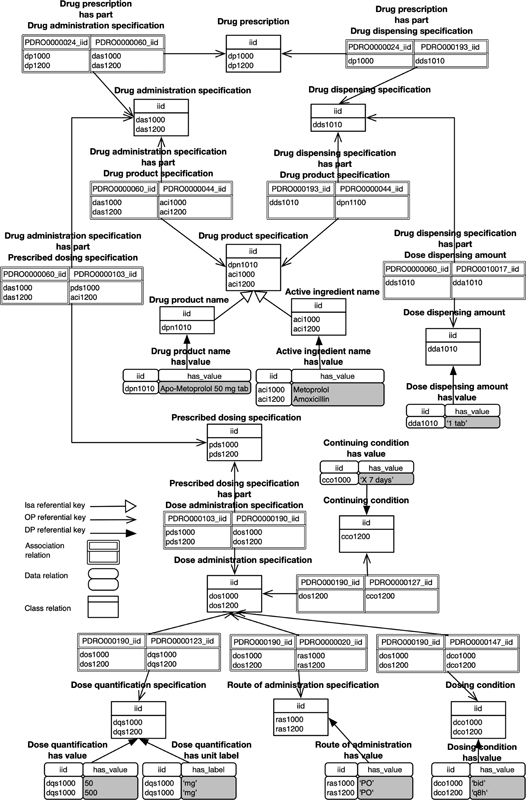
OntoRel with data as a relation schema.

These issues are solved using a relational data model generated from an ontology because it provides the explicit semantic of the data. The data model is illustrated in two forms: the graph form ( Fig. 10 ) and the relational form ( Fig. 11 ). Fig. 10 illustrates, as a graph, part of the generated relational data model from PDRO with data examples. The full rectangles represent a data relation containing tuples. The dotted rectangles represent a class relation with calculated tuples. The lines represent the association relations.

Fig. 11 illustrates part of the generated relational data model from PDRO with data examples.

Finally, for other use cases, the relational data model can be used as a target model to store data extracted from natural language processing or for classification using ontology reasoners.

## Related Work

The literature suggested several methods to derive a relational data model from an ontology. A literature review was conducted in mid-2018 to explore methods published after 2010 describing RDB generation from an ontology. 17 A list of 23 criteria was defined to evaluate and compare the 10 most relevant papers.

The present article extends this review with recent publications and criterion coverage details (see Appendix A). The evaluated papers: A1.Dou.2010, 18 A2.Bellatreche.2010, 19 A3.Saccol.2011, 20 A4.Vyšniauskas.2012, 21 A5.Hornung.2013, 22 A6.Podsiadły-Marczykowska.2014, 23 A7.Jiménez-Ruiz.2015, 24 A8.Ho.2015, 25 A9.Afzal.2016, 26 A10.Achpal.2016, 27 A11.Mahmudi.2018 28 and A12.Guidoni.2020. 29

The evaluated methods differ in several ways: the ontology constructs considered during the conversion, the completeness of the generated relational model, and the availability of tools that implement the method. As expected, all the methods convert a class into a relation with a primary key. Regarding axioms, complex ones are never considered nor handled, which is a significant issue for biomedical ontologies. Simple ones follow two dominant approaches: they are converted into an attribute or into a relation. Converting an axiom into an attribute may introduce several issues, such as missing information (nulls) for zero-to-one or zero-to-many relationships and data redundancy for many-to-many relationships. Converting an axiom into a relation may impact the query performance, but this is a better approach to guarantee data integrity and structure extension, as performance can be handled at the physical level by the RDB management systems using adequate indexing structure. 30 These axiom conversions and the inability to deal with complex axioms reduce the structural uniformity across all the relations in the data model and increase semantic loss.

Moreover, the review outlined limitations in different areas which may lead to semantic loss, including the lack of conversion rules for object properties, property inheritance axioms, cardinality restriction, property characteristics, and annotation, as well as the inability to process complex axioms (set of simple expressions linked with logical operators) that are widely used in biomedical ontologies. Most methods store some important ontological constructs into metadata tables and do not derive explicit structure or constraints from them. As a result, the axiom verification will be incomplete or challenging to automate. Thus, the main challenge is maintaining the richness of ontological definitions in the resulting relational data model by converting uniformly and consistently ontology constructs into relational constructs to detect the data that do not conform to the axioms.

Finally, only two implementations are publicly available, the first one is accessible through a web page, 22 but the source code is not available to be used and extended by other researchers. The second works with ontology defined using OntoUML based on UFO (Unified Foundational Ontology) 29 and allows defining ontologies using a graphical interface. However, defining and maintaining large biomedical ontologies using a graphical interface are not always convenient. Therefore, a more generic method is needed to benefit from the mature ontologies already in the OBO Foundry.

## Contribution and Future Work

This work aimed to address the problem of designing a sharable and interoperable relational data model not only to store data coming from heterogeneous systems but also to store the associated semantic to support various applications. The method described in this article offers an advanced conversion process to enable the automatic generation of the uniform relational data model with constraints to maximize semantic preservation and control data integrity. More specifically, the presented method differs from the existing ones by the following features:

- The axiom complexity reduction allows a concise and uniform conversion.
- The normalization of the data model increases automation and uniformity.
- The generation of advanced constraints such as quantification, intersection, and union using functions increases data integrity control.
- The transformation of ontological annotations into SQL comments and views provides documentation and multiple access points within the data model.
- The configurable transformation of OWL types into SQL types allows a uniform usage of OWL types.
- The generation of a mapping dictionary enables structure reversibility and data extraction in various output formats.
- The implementation, OntoRelα, can be used with various OWL ontologies and PostgreSQL databases.

However, several limitations will require further work. First, the relatively large size of the resulting relational data model is a challenge, especially when trying to interact manually with the model. As an example, to get all the data related to a drug prescription, we need to build a query with at least 16 joints (see Fig. 11 ). This limitation could be alleviated by using navigation tools and query builder applications fully leveraging this new approach to benefit from the full semantic while helping the user find the needed construct faster. Second, structural redundancy in the data model is caused by redundant axioms. Advanced axiom redundancy reduction rules are already under development to address this, yielding a smaller RDB while fully preserving its semantic. Finally, more conversion rules are being defined to improve the data integrity and data ingestion, such as deriving secondary keys, general constraints using property characteristics, 24 27 and generating data modification procedures 27 to ensure data quality.

## Conclusion

Many conversion methods from an ontology to a relational data model have already been proposed. However, these propositions suffer from limitations regarding the coverage of ontological constructs, the handling of complex axioms, and the accessibility of tools. This article presented conversion rules and a freely available prototype named OntoRelα that covers more ontological constructs and handles complex axioms, and that can be used and extended by other researchers. This work is a first step toward building a tool to generate a sharable and interoperable database using ontologies. More development is underway to refine the derived relational data model and provide complementary data access tools.

**Table 1:** Criterion application by method [fulfilled (X), not fulfilled (–), unknown(?)] a

	A1 18	A2 19	A3 20	A4 21	A5 22	A6 23	A7 24	A8 25	A9 26	A10 27	A11 28	A12 29	OntoRelα
Ontology													
Ontology language	?	?	?	?	X	X	X	?	X	X	?	–	X
Schema													
Structure	?	X	–	X	X	–	X	?	?	?	?	X	X
Domains	–	–	–	–	–	–	–	–	–	–	–	–	X
Primary keys	X	X	X	X	X	X	X	X	X	X	?	X	X
Secondary keys	–	?	–	X	?	?	X	?	?	X	?	–	X
Foreign keys	?	X	X	X	X	X	X	X	X	X	X	X	X
Participation constraint	?	?	–	–	–	–	X	–	–	X	?	?	X
General constraint	?	?	X	X	?	X	X	X	–	X	?	X	–
Modification procedure	–	–	–	–	–	–	–	–	–	X	?	–	–
Target DBMS	?	?	X	?	?	X	?	X	X	?	?	?	X
Process													
Axiom reduction	?	?	–	?	?	–	–	?	?	?	–	?	X
Intermediate structure	–	X	–	X	–	–	?	X	X	?	?	X	X
Type conversion	?	?	–	?	?	?	X	X	X	?	?	?	X
Restriction conversion	?	X	–	X	–	–	X	X	X	X	?	?	X
Individual conversion	X	–	–	–	X	–	X	X	–	X	X	?	–
Annotation conversion	–	–	–	X	X	–	–	–	–	–	?	–	X
Structural reversibility	X	X	–	X	–	?	–	X	X	?	–	?	X
Tuple reversibility	X	X	–	X	X	?	X	?	?	?	–	?	–
Tool													
Implementation	X	X	X	X	–	X	X	X	X	?	X	X	X
Availability	–	?	?	–	X	?	–	?	?	?	?	X	X
*Total (X)*	*5*	*7*	*5*	*10*	*8*	*6*	*12*	*9*	*8*	*9*	*9*	*7*	*16*

a Extended with permission from Khnaisser C, Lavoie L, Burgun A, Ethier JF. Generating relational database using ontology review: issues, challenges and trends. Int J Adv Comput Sci Appl. 2018;9(6):139–145.

## Appendices

## Appendix A

This section presents the 23 criteria defined to evaluate and compare the 12 most relevant papers against our method.

### Criterion Application by Method

Table 1 presents the comparison criteria applicable to each compared paper. A criterion is fulfilled by the method (X), not fulfilled (–), or unknown (?). For example, for A1, the criterion “Ontology language” is unknown, “Domains” are not fulfilled, and “Primary Keys” are fulfilled. Also, the count of fulfilled criteria is calculated for each method. *OntoRelα* fulfills 16 criteria over 23. To address the remaining criteria, this article introduces an advanced analysis of the ontology (1) to extract property characteristics and datatype constraints, (2) to generate modification procedures for data that verify integrity constraints, and (3) to implement the individual and tuple reversibility algorithm.

The evaluated papers: A1.Dou.2010, 18 A2.Bellatreche.2010, 19 A3.Saccol.2011, 20 A4.Vyšniauskas.2012, 21 A5.Hornung.2013, 22 A6.Podsiadły-Marczykowska.2014, 23 A7.Jiménez-Ruiz.2015, 24 A8.Ho.2015, 25 A9.Afzal.2016, 26 A10.Achpal.2016, 27 A11.Mahmudi.2018 28 and A12.Guidoni.2020 29 .

### Criteria Definition

Here is the list of criterion definitions used to compare each method:

- Ontology
  – Ontology language—the ontology language supported by the method: OWL-DL, OWL-QL, OWL-RL, OWL- EL, RDF(S), DAML, etc.
- Schema
  – Structure—the relational schema normal form: 3NF, BCNF, 5NF, or 6NF. This criterion can be deduced from the conversion rules.
  – Domains—does the method convert the ontology data types and their constraints into domains (e.g., CREATE DOMAIN in PostgreSQL)?
  – Primary keys—does the method generate [G] or calculate [C] the primary keys?
  – Secondary keys—does the method generate the secondary key from the set of axioms?
  – Foreign keys—does the method convert the appropriate axioms into foreign keys?
  – Participation constraints—does the method convert cardinalities into constraints?
  – General constraints—does the method convert datatype and disjoint constraints into general constraints?
  – Modification procedures—does the method define the procedures for modifying the data (insert, delete, and update triggers)?
  – Target RDBMS—such as PostgreSQL, MySQL, Oracle, MSSQL, etc.
- Process
  – Axiom reduction—does the conversion process deal with complex axioms?
  – Intermediate structure—the intermediate data structure used to convert OWL into a relational schema: MOF (Meta-Object Facility), FOL (First-order logic), RDF, Jena model, etc.
  – Type conversion—does the conversion process specify or configure the conversion rules between ontology types and SQL types?
  – Restriction conversion—does the conversion process convert the restrictions to general constraints?
  – Annotation conversion—does the conversion process convert the annotations to document the relational schema?
  – Structural reversibility—does the conversion process make it possible to refer to the ontology construct? Furthermore, does the method describe the algorithm and propose an implementation of structural reversibility?
  – Tuple reversibility—does the conversion process make it possible to import tuples stored in the DB in their full ontological expression? Furthermore, does the method describe the algorithm and propose an implementation of tuple reversibility?
- Tool
  – Implementation—has the method been implemented?
  – Availability—is the tool publicly available?

## Appendix B

Each expression in an axiom is syntactically analyzed using the abstract grammar and reduced recursively according to reduction rules until a set of simple axioms is reached.

axiom::= domain operator range
domain::= expression
range::= expression
expression::= ID
| expression ('⊔' | '⊓') expression
| property
property::= '(' operator expression ')'
operator::= '⊑' | propertyOperator | qt
propertyOperator::= ID
qt::= '[' N '..' N ']'
N::= /* positive or null integer */
ID::= /* an identifier (e.g., IRI)*/

The reduction is achieved through the function Ф with three components: Ф _1_ , Ф _2_ , and Ф _3_ . All the components take as an input an axiom.

- Ф _1_ returns the class representing the concept (it can be the same class or a class not defined in the original ontology).
- Ф _2_ returns a set of constraints that must be guaranteed to preserve the semantic validity.
- Ф _3_ returns a set of new reduced axioms produced by Ö that replaces the input axiom.

The reduction function Ф is defined by recursion regarding the four types of expressions of an axiom as follows:



where

- A is an ID of a class.
- Ź is the ID new generated new class.
- *β* and *ᵧ* are expressions as defined in the grammar.

Therefore, the reduction produces a set of new classes, axioms, and constraints calculated by Ф. The new axioms are all derivable by the grammar and have the following final simple form {axiom::= ID operator ID}.

## Appendix C

**Fig. 12:**
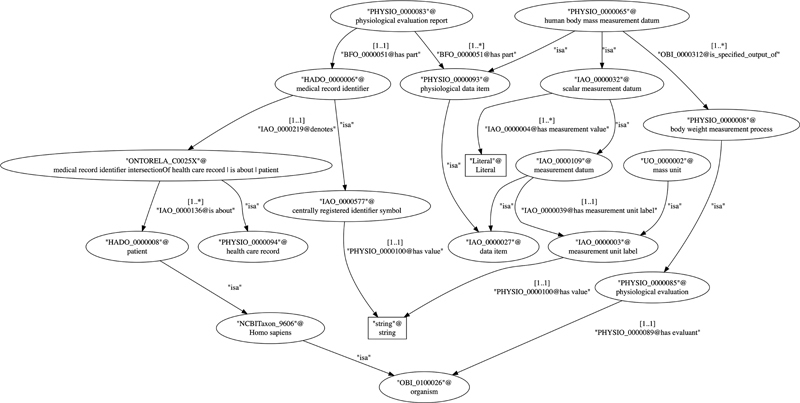
The ontology graph derived from a sample of physiological measurement ontology.

**Fig. 13:**
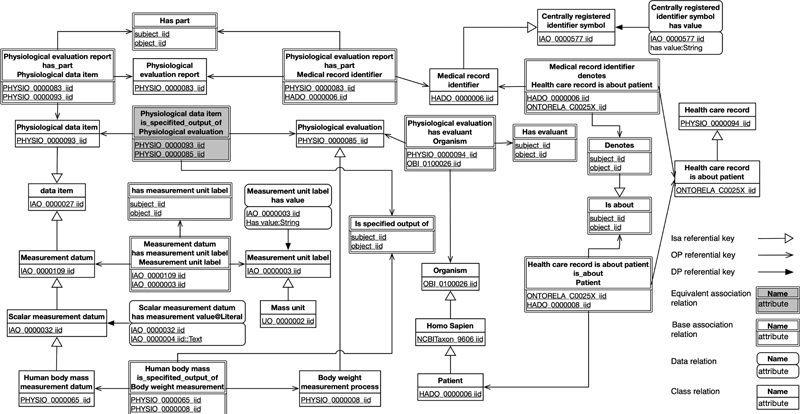
The relational data model derived from a sample of physiological measurement ontology.

The example in Figure 12 shows a sample of physiological measurement ontology, the human body mass. The code below presents some ontological constructs in a simplified syntax, and the figures below, Figure 12 and Figure 13 , illustrate the generated relational data model as a graph and as a relation schema respectively. The complete example can be found on GitHub: https://github.com/OpenLHS/OntoRela .

## Notes

OP “has part”
OP “has measurement unit label”
DP “has specified numeric value”
CLASS “Rate of administration specification”
ISA “Information content entity”
**“has part” [1..1] (“Mass per time value specification” OR “Volumetric flow rate value specification”)**
CLASS “Mass per time value specification”
ISA “Scalar value specification”
“has measurement unit label” [1..1] “Mass per time unit”
“has specified numeric value” [1..1] “positive rational”
CLASS “Volumetric flow rate value specification”
ISA “Scalar value specification”
“has measurement unit label” [1..1] ““Volumetric flow unit”
“has specified numeric value” [1..1] “positive rational”
CLASS “Volumetric flow unit” “has part” [1..1] “Mass unit” “has part” [1..1] “Time unit”
CLASS “Mass per time unit” “has part” [1..1] “Volume unit” “has part” [1..1] “Time unit”
CLASS “Mass unit”
ISA “Measurement unit label”
“has value” [1..1] String
CLASS “Time unit”
ISA “Measurement unit label”
“has value” [1..1] String
CLASS “Volume unit”
ISA “Measurement unit label”
“has value” [1..1] String

## Notes

**CLASS “Mass per time value or Volumetric flow rate value specification”**
CLASS “Rate of administration specification”
ISA “Information content entity”
**“has part” [1..1] “Mass per time value or Volumetric flow rate value specification”**
CLASS “Mass per time value specification”
ISA “Scalar value specification”
**ISA “Mass per time value or Volumetric flow rate value specification”**
“has measurement unit label” [1..1] “Mass per time unit”
“has specified numeric value” [1..1] “positive rational”
CLASS “Volumetric flow rate value specification”
ISA “Scalar value specification”
**ISA “Mass per time value or Volumetric flow rate value specification”**
“has measurement unit label” [1..1] “Volumetric flow unit”
“has specified numeric value” [1..1] “positive rational”

## Notes

ONTOLOGY”:humanbodyweight-example.owl”
PROPERTY”:BFO_0000050” LABEL @en 'part of'
PROPERTY”:BFO_0000051” LABEL @en 'has part'
PROPERTY: “IAO_0000039” LABEL @en 'has measurement unit label'
ISA”:BFO_0000051”
PROPERTY”:IAO_0000136” LABEL @en 'is about'
PROPERTY”:IAO_0000219” LABEL @en 'denotes'
ISA”:IAO_0000136”
PROPERTY”:OBI_0000299” LABEL @en 'has_specified_output'
PROPERTY”:OBI_0000312” LABEL @en 'is_specified_output_of'
PROPERTY: “OBI_0001938” LABEL @en 'has value specification'
PROPERTY”:RO_0000057” LABEL @en 'has participant'
PROPERTY”:PHYSIO_0000089” LABEL @en 'has evaluant'
ISA”:RO_0000057”
PROPERTY”:IAO_0000004” LABEL @en 'has measurement value'
PROPERTY”:PHYSIO_0000100” LABEL @en 'has value'
CLASS”:PHYSIO_0000083”LABEL @en'physiological evaluation report'
”:BFO_0000051” [1..1]”:HADO_0000006”
”:BFO_0000051” [1..*]”:PHYSIO_0000093”
CLASS”:PHYSIO_0000085” LABEL @en 'physiological evaluation'
”:PHYSIO_0000089” [1..1]”:OBI_0100026”
CLASS”:PHYSIO_0000093” LABEL @en 'physiological data item'
ISA”:IAO_0000027”
CLASS”:PHYSIO_0000008” LABEL @en 'body weight measurement process'
ISA”:PHYSIO_0000085”
CLASS”:PHYSIO_0000065” LABEL@en'human body mass measurement datum'
ISA”:IAO_0000032”
ISA”:PHYSIO_0000093”
”:OBI_0000312” [1..*]”:PHYSIO_0000008”
CLASS”:IAO_0000027” LABEL @en 'data item'
CLASS”:IAO_0000109” LABEL @en 'measurement datum'
ISA”:IAO_0000027”
”:IAO_0000039” [1..1]”:IAO_0000003”
CLASS”:IAO_0000032” LABEL @en 'scalar measurement datum'
ISA”:IAO_0000109”
”:OBI_0001938” [1..*]”:OBI_0001931”
”:IAO_0000004” [1..1]”:double”
CLASS”:OBI_0001931” LABEL @en 'scalar value specification'
CLASS”:IAO_0000003” LABEL @en 'measurement unit label'
”:PHYSIO_0000100” [1..1]”:string”
CLASS”:UO_0000002” LABEL @en 'mass unit'
ISA”:IAO_0000003”
CLASS”:HADO_0000008” LABEL @en 'patient'
ISA”:NCBITaxon_9606”
CLASS”:HADO_0000006” LABEL @en 'medical record identifier'
”:IAO_0000219” [1..1]”:ONTORELA_C0025X”
CLASS”:OBI_0100026” LABEL @en 'organism'
CLASS”:NCBITaxon_9606” LABEL @en 'Homo sapiens'
ISA: “OBI_0100026”
CLASS”:ONTORELA_C0025X”
LABEL @en ' health care record is about patient'
ISA”:PHYSIO_0000094”
”:IAO_0000136” [1..*]”:HADO_0000008”
CLASS”:PHYSIO_0000094” LABEL @en 'health care record'
